# A method to assess the clinical significance of unclassified variants in the *BRCA1 *and *BRCA2 *genes based on cancer family history

**DOI:** 10.1186/bcr2223

**Published:** 2009-02-06

**Authors:** Encarna B Gómez García, Jan C Oosterwijk, Maarten Timmermans, Christi J van Asperen, Frans BL Hogervorst, Nicoline Hoogerbrugge, Rogier Oldenburg, Senno Verhoef, Charlotte J Dommering, Margreet GEM Ausems, Theo AM van Os, Annemarie H van der Hout, Marjolijn Ligtenberg, Ans van den Ouweland, Rob B van der Luijt, Juul T Wijnen, Jan JP Gille, Patrick J Lindsey, Peter Devilee, Marinus J Blok, Maaike PG Vreeswijk

**Affiliations:** 1 Department of Clinical Genetics, University Hospital Maastricht, PO Box 5800, 6202 AZ Maastricht, the Netherlands; 2 Department of Genetics and Cell Biology, Maastricht University Medical Center, Research Institute Growth & Development, P. Debyelaan 25, Maastricht 6229 HX, the Netherlands; 3 Department of Genetics, University Medical Center, Groningen University, Hanzeplein 1, Groningen 9713 GZ, the Netherlands; 4 Center for Human and Clinical Genetics, LUMC, Albinusdreef 2, Leiden 2333 ZA, the Netherlands; 5 Antoni van Leeuwenhoek Hospital, Plesmanlaan 121, Amsterdam 1066 CX, the Netherlands; 6 Department of Human Genetics, Radboud University Nijmegen Medical Center, Geert Grooteplein Zuid 8, Nijmegen 6525 GA, the Netherlands; 7 Department of Clinical Genetics, Erasmus Medical Center, Westzeedijk 112, Rotterdam 3016 AH, the Netherlands; 8 Department of Clinical Genetics, VU University of Amsterdam Hospital, De Boelelaan 1117, Amsterdam 1081 HV, the Netherlands; 9 Department of Medical Genetics, University Medical Center Utrecht, Heidelberglaan 100, Utrecht 3584 CX, the Netherlands; 10 Department of Genetics, Academic Medical Center in Amsterdam, Meibergdreef 9, Amsterdam 1105 AZ, the Netherlands

## Abstract

**Introduction:**

Unclassified variants (UVs) in the *BRCA1/BRCA2 *genes are a frequent problem in counseling breast cancer and/or ovarian cancer families. Information about cancer family history is usually available, but has rarely been used to evaluate UVs. The aim of the present study was to identify which is the best combination of clinical parameters that can predict whether a UV is deleterious, to be used for the classification of UVs.

**Methods:**

We developed logistic regression models with the best combination of clinical features that distinguished a positive control of *BRCA *pathogenic variants (115 families) from a negative control population of *BRCA *variants initially classified as UVs and later considered neutral (38 families).

**Results:**

The models included a combination of BRCAPRO scores, Myriad scores, number of ovarian cancers in the family, the age at diagnosis, and the number of persons with ovarian tumors and/or breast tumors. The areas under the receiver operating characteristic curves were respectively 0.935 and 0.836 for the *BRCA1 *and *BRCA2 *models. For each model, the minimum receiver operating characteristic distance (respectively 90% and 78% specificity for *BRCA1 *and *BRCA2*) was chosen as the cutoff value to predict which UVs are deleterious from a study population of 12 UVs, present in 59 Dutch families. The p.S1655F, p.R1699W, and p.R1699Q variants in *BRCA1 *and the p.Y2660D, p.R2784Q, and p.R3052W variants in *BRCA2 *are classified as deleterious according to our models. The predictions of the p.L246V variant in *BRCA1 *and of the p.Y42C, p.E462G, p.R2888C, and p.R3052Q variants in *BRCA2 *are in agreement with published information of them being neutral. The p.R2784W variant in *BRCA2 *remains uncertain.

**Conclusions:**

The present study shows that these developed models are useful to classify UVs in clinical genetic practice.

## Introduction

Cancer risk counseling of patients and families with an unclassified variant of the breast cancer (BC) genes *BRCA1 *and/or *BRCA2 *(MIM numbers 113705 and 600185, respectively) has become a prominent issue for genetic counselors and oncologists. About one-third of the genetic variants in *BRCA1 *and 50% of those found in *BRCA2 *reported by the Breast Cancer Information Core [1] are considered genetic variants of unknown clinical significance, also known as unclassified variants (UVs), because of the uncertainty about their cancer risks. This is often the case for missense variations or when the nucleotide change affects or creates a (putative) splice-site.

As opposed to the families with deleterious variants – where asymptomatic relatives can be offered DNA diagnosis, and carriers are eligible for risk-reducing interventions and/or surveillance – presymptomatic testing is not possible in families with a UV, and surveillance can only be based upon the severity of the cancer family history.

In addition to biochemical and epidemiological criteria [2-5], information about co-segregation studies, co-occurrence with a deleterious variant [6,7], loss of heterozygosity in the tumor [8], histopathologic characteristics [9,10], and functional assays [11,12] have been used to classify UVs. Several comprehensive models have been published that use combinations of the above-mentioned parameters [6,7,9,12-14]. Limitations of those models can be that some of the parameters included are not always available or are only suitable for missense variants but not for other types of UVs.

Even though quantification of BC and/or ovarian cancer (OC) events in the families is easy to record and is the most direct sign of clinical relevance, cancer family history has only rarely been used to classify UVs [14,15]. In a previous study [15] we found that patients with a UV have, as a group, significantly lower *a priori *scores using the BRCAPRO [16] and Myriad [17] models than patients with a pathogenic variant. More recently, Easton and colleagues have provided multifactorial logistic regression models to classify UVs in the *BRCA *genes [14]. Those models include information about the proband (that is, disease status and age of diagnosis) and family history, which is categorized into *n *types, according to the number of relatives with cancer (BC or OC) and the age of diagnosis. The estimated likelihood ratio is combined with the likelihood ratios obtained from the other two components of the models – co-occurrence *in trans *with a known deleterious mutation and co-segregation – to provide a global assessment for each UV. This approach, which uses those parameters most directly associated with the clinical outcome, has recently been extended to UVs in other cancer genes [18].

In the present study, we have elaborated logistic regression models using the most discriminative clinical features that distinguish between deleterious and neutral variants in *BRCA1 *and *BRCA2*. Subsequently, we have applied them to a group of 12 UVs found in 59 Dutch families with BC and/or OC.

## Materials and methods

### Subjects

All of the probands from the families included in the study had been selected for DNA diagnosis of *BRCA1 *and *BRCA2*, according to the same selection criteria defined by a group of experts and used nationwide. These criteria are based on the number of first-degree and/or second-degree relatives with BC and/or OC, and the age of diagnosis. Each of the indication criteria corresponds with at least a 10% chance of finding a mutation in those genes.

#### Control populations

Families diagnosed and counseled at the Academic Medical Center of Maastricht were used as controls. Table 1 presents the clinical parameters evaluated in the control populations.

**Table 1:** Descriptive statistics

	Pathogenic variants (positive controls)		Neutral variants (negative controls)		*P *value
	
	Number of probands	Mean ± standard deviation/percentage	Number of probands	Mean ± standard deviation/percentage	
Proband data					
BRCAPRO score all^a^	115	0.580 ± 0.353	38	0.381 ± 0.219	0.000
BRCAPRO1 score^a^	65	0.472 ± 0.300	20	0.293 ± 0.155	0.000
BRCAPRO2 score^a^	50	0.244 ± 0.186	18	0.163 ± 0.123	0.001
Myriad II score^a^	115	0.241 ± 0.145	38	0.174 ± 0.094	0.000
Sex (male)^b^	115	3.5	38	0	0.000
BC^b^	115	80.9	38	84.2	0.810
Bilateral BC^b^	115	14.8	38	18.4	0.611
Age at BC diagnosis^c^	93	43.419 ± 10.208	32	46.178 ± 9.591	0.008
OC^b^	115	18.3	38	5.3	0.066
Age at OC diagnosis^c^	21	54.381 ± 9.967	2	48.190 ± 9.899	0.108
Family data					
Members affected^d^	115	3.548 ± 1.640	38	3.234 ± 1.124	0.056
Proportion affected^a^	115	0.183 ± 0.094	38	0.173 ± 0.078	0.199
Number of tumors^d^	115	4.200 ± 1.957	38	3.732 ± 1.329	0.007
Total BC tumors^d^	115	3.400 ± 2.021	38	3.266 ± 1.277	0.422
Bilateral BC^d^	115	0.435 ± 0.637	38	0.375 ± 0.620	0.279
BC in males^b^	115	10.4	38	2.6	0.820
Age at BC diagnosis^c^	115	45.607 ± 7.951	38	48.623 ± 7.577	0.000
Total OC tumors^d^	115	0.800 ± 0.929	38	0.466 ± 0.343	0.000
Age at OC diagnosis^c^	64	52.982 ± 10.323	5	51.091 ± 11.234	0.435

BC, breast cancer; OC, ovarian cancer. ^a^Univariate gamma linear regression model (*t *test). ^b^Two-by-two table (Fisher's exact test). ^c^Univariate Gaussian linear regression model (*t *test). ^d^Univariate Poisson linear regression model (*z *test).

**Figure 1:**
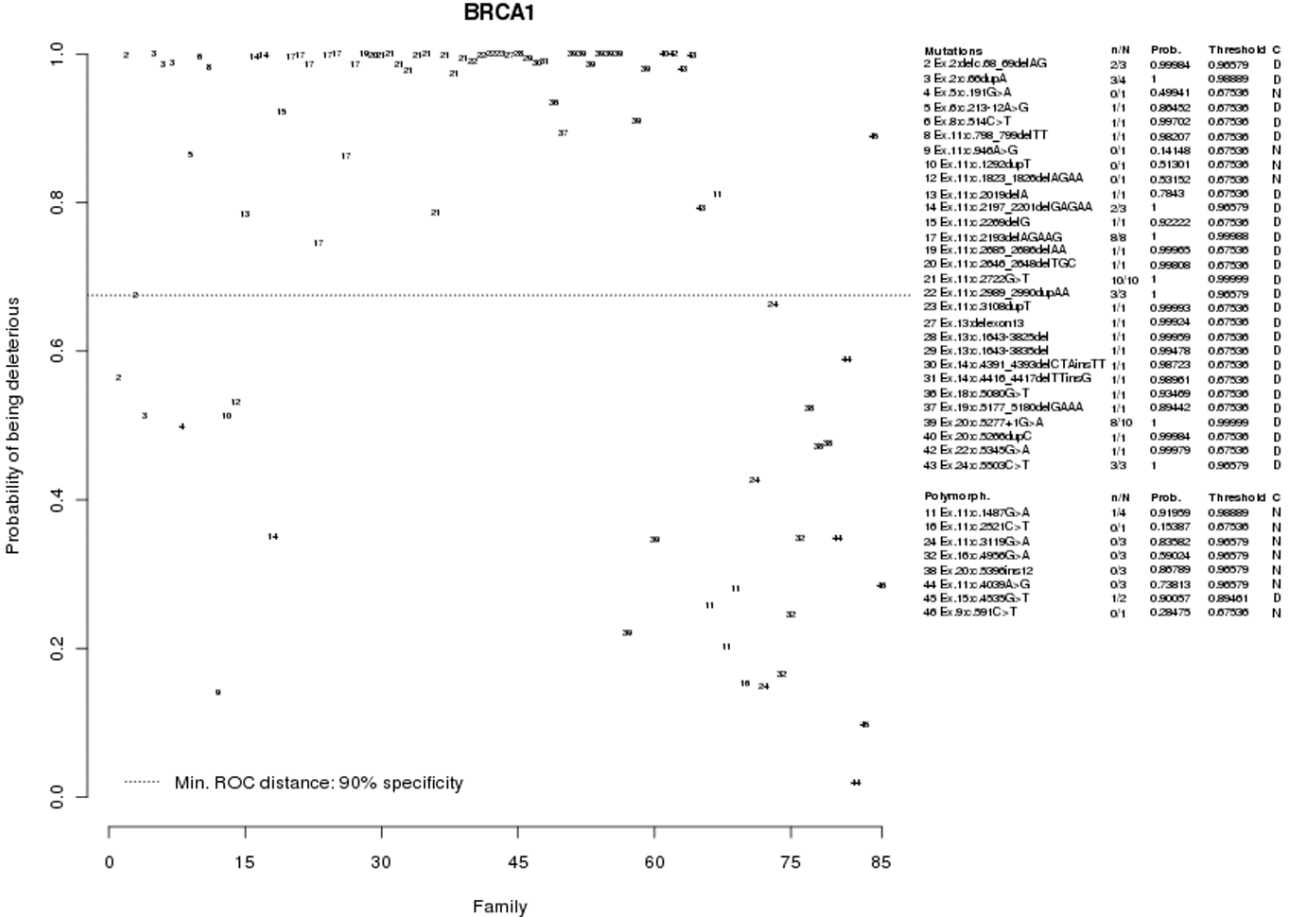
Predicted probabilities of the *BRCA1 *control populations. Plot showing the predicted probabilities of the control populations – deleterious (mutations) and neutral variants (polymorphisms) – in *BRCA1 *using the logistic regression model obtained for *BRCA1*. Dotted cutoff lines, probability from the *BRCA1 *model that minimizes the receiver operating characteristic (ROC) distance. For each genetic variant, the number of families above the cutoff point and the total number of families (*n*/*N*) is presented on the right side along with the probability of having at least one correct prediction (Prob.) and the probability if all families of the genetic variant under consideration were on the cutoff point (threshold). Finally, the classification (C) as a deleterious variant (D) or not known (N) is also presented. Sequence nomenclature: NCBI reference sequence U14680.1 (*BRCA1*), numbering starting at the A of the ATG translation initiation codon.

**Figure 2:**
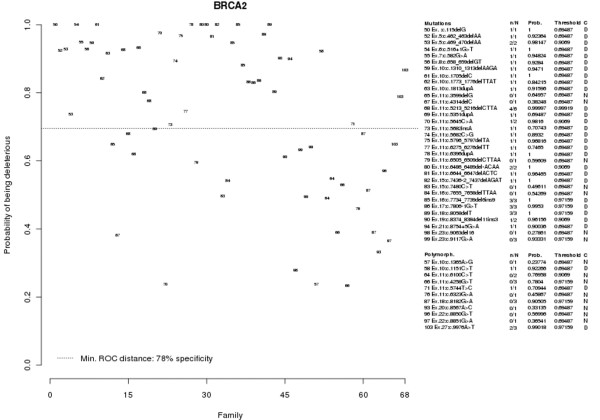
Predicted probabilities of the *BRCA2 *control populations. Plot showing the predicted probabilities of the control populations in *BRCA2 *using the logistic regression model obtained for *BRCA2*. Dotted cutoff lines, probability from the *BRCA2 *model that minimizes the receiver operating characteristic (ROC) distance. The parameters evaluated for the *BRCA1 *variants (explained in Figure 1) are also shown for each of the *BRCA2 *genetic variants. Sequence nomenclature: NCBI reference sequence U43746.1 (*BRCA2*), numbering starting at the A of the ATG translation initiation codon.

The positive control group consisted of 115 unrelated probands with a deleterious variant (65 with a variant in *BRCA1 *and 50 with a variant in *BRCA2*) and included 62 different mutations (29 mutations in *BRCA1 *and 33 mutations in *BRCA2*). The negative control group consisted of 38 index cases (20 cases in *BRCA1 *and 18 cases in *BRCA2*) with 19 different genetic variants (eight with a variant in *BRCA1 *and 11 with a variant in *BRCA2*) that were initially classified as UVs, but later were considered neutral variants. For a detailed description of the sequence variants included, we refer to Figures 1 and 2.

The total numbers of first-degree and second-degree relatives in each of the control populations were 2,619 in the deleterious (positive control) population and 798 in the neutral variant (negative control) populations.

#### Study population: patients with an unclassified variant

Each diagnostic laboratory in the Netherlands selected five UVs in either *BRCA1 *or *BRCA2 *that were of particular interest for that center (for example, multiple families with the same UV, large families in which the UV was segregating). A list was made that contained all of the selected UVs. From this list was made a shortlist with those UVs that were present in several centers and/or met at least one of the following criteria: Grantham score >100 [19]; the UV is located within a structural domain (for example, BRCA C-terminal region domains in *BRCA1 *or the BRC repeats in *BRCA2*) or in a domain that is necessary for interaction with other proteins (for example, RAD51 binding sites in *BRCA1 *and *BRCA2*) [20]; and the amino acid change has a high degree of evolutionary conservation (that is, invariant through *Tetraodon nigroviridis*) [21].

UVs co-occurring with a *BRCA *deleterious variant in the proband were excluded. In addition, priority was given to those UVs found in more than one family and/or genetic center.

The UVs selected (four in *BRCA1 *and eight in *BRCA2*) were: *BRCA1*: ex.11:c.736T>G (p.L246V), ex.16:c.4964C>T (p.S1655F), ex.18:c.5095C>T (p.R1699W), and ex.18:c.5096G>A (p.R1699Q); and *BRCA2*: ex.3:c.125A>G (p.Y42C), ex.10:c.1385A>G (p.E462G), ex.18:c.7978T>G (p.Y2660D), ex.19:c.8350G>A (p.R2784W), ex.19:c.8351C>T (p.R2784Q), ex.21:c.8662C>T (p.R2888C), ex.24:c.9154C>T (p.R3052W), and ex.24:c.9155G>A (p.R3052Q). The nomenclature used for the description of the sequence variations is that according to the Human Genome Variation Society [22].

Table 2 summarizes the available biochemical and epidemiological data of the UVs. Please note that the p.L246V (*BRCA1*) and the p.E462G (*BRCA2*) variants did not meet any of the criteria mentioned above, but were selected because those UVs were found in more than one genetic center. During the course of our study, the p.R1699W in *BRCA1 *was reclassified as pathogenic in the Breast Cancer Information Core [1].

**Table 2:** The unclassified variants in the present study: epidemiological and biochemical criteria

Variant [22]	Number of families	Co-segregation (present study)^a^	Polarity change	Conserved mammals/other [21]^b^	Grantham score [19]	Times reported (Breast Cancer Information Core [1])	Co-segregation (literature)	Co-occurrence (literature)	Classification (literature)	Reference^c^
*BRCA1*										
p.L246V	2	ND	No	No/No	32	79	No	Yes (several)	Neutral	[5,7,13]
p.S1655F	2	6/6 (n = 2)	Yes	Yes/Yes	155	3	Not done	Not done	Deleterious	[5,32,34,35]
p.R1699W	9	8/8 (n = 4)	Yes	Yes/Yes	101	13	Not done	No	Deleterious	[1,14,35]
p.R1699Q	5	1/2 (n = 2)^d^	Yes	Yes/Yes	43	11	Yes	No	Deleterious, uncertain, low/moderate	[5,6,9,12,32,34,35]
Total	18									
*BRCA2*										
p.Y42C	3	ND	N	No/No	194	14	No	Yes	Neutral	[6,9,11]
p.E462G	8	2/5 (n = 5)	Yes	Yes/No	98	35	No	Yes (Y3097X)	Neutral	[11,12]
p.Y2660D	9	8/8 (n = 5)	Yes	Yes/Yes	160	2	Not done	Not done		None
p.R2784W	1	ND	Yes	Yes/Yes	101	5	Not done	Not done	Uncertain	[33]
p.R2784Q	4	1/2 (n = 2)^e^	Yes	Yes/Yes	43	4	Not done	Not done		None
p.R2888C	5	1/1 (n = 1)	Yes	No/Yes	180	4	No	Yes	Neutral	[14]
p.R3052W	10	1/1 (n = 1)	Yes	Yes/Yes	101	8	Not done	Not done	Deleterious	[33]
p.R3052Q	1	0/1 (n = 1)	Yes	Yes/Yes	43	3	Not done	Yes	Neutral	[14]
Total	41									

^a^Co-segregation in the present study is expressed as number of tested positive (proband excluded)/total number of affected relatives tested (n = number of families tested). ^b^Alignments based on the following species and NCBI reference sequences: *BRCA1*: human (NP_009225), chimp (AAG43492), gorilla (AAT44835), orang (AAT44834), macacque (AAT44833), dog (AAC48663), mouse (AAD00168), cow (NP848668), opossum (AAX92675), chicken (NP989500), xenopus (AAL13037), and pufferfish (AAR89523); and *BRCA*2: human (NP000050), chimp (XP509619), dog (BAB91245), mouse (AAB48306), chicken (AAL89470), and tetraodon (CAG09009). ^c^Functional studies were performed in [11,12,32-35]. ^d^Lack of co-segregation was observed in one of the two pedigrees analyzed. In that pedigree, the proband had BC at age 37, her father's sister had BC at age 61, her cousin (the daughter of that aunt) had BC at age 45 – did not have the UV. ^e^This UV does not co-segregate with the disease in one family, which consisted of two affected members: the proband had BC at age 31, her mother with BC at age 67 – did not have the UV.

All patients included in this study gave informed consent and the study was approved by the Medical Ethical Committees of the medical centers.

The same clinical parameters analyzed in the control population were also collected in the families of the study population. The total number of first-degree and second-degree relatives was 1,082.

### Laboratory diagnosis

*BRCA1 *and *BRCA2 *were analyzed from blood samples by denaturing high-performance liquid chromatography. Additional technical details, primers and denaturing high-performance liquid chromatography elution profiles are available from the authors upon request. Changes in denaturing high-performance liquid chromatography elution profiles were verified by standard sequence analysis. Until 10 years ago, a protein truncation test was used to analyze exon 11 of *BRCA1 *and exons 10 and 11 of *BRCA2*. In those cases, the rest of the gene was more recently fully analyzed by denaturing high-performance liquid chromatography. In addition, multiplex ligation-dependent probe amplification analysis was performed for *BRCA1 *to detect large duplications or deletions.

### Statistical analysis

The BRCAPRO and Myriad models are distributed as a part of the counseling package CancerGene from the U.T. Southwestern Medical Center at Dallas [16,17].

BRCAPRO [16] is a Mendelian model that incorporates mutated allele frequencies and cancer-specific penetrances, in addition to the following clinical parameters about the proband and the first-degree and second-degree relatives: the number of women affected with BC only; the number of women affected with OC only; discrimination between paternal/maternal inheritance patterns; BC under age 50 and OC (any age); bilateral BC; a relative with both OC and BC; affected and unaffected individuals; Ashkenazi Jewish ancestry; and male BC.

The Myriad II prevalence tables [17] are based on proband and family history accompanying results of *BRCA1 *and *BRCA2 *deleterious variant samples tested by the company. Unlike the BRCAPRO model, these tables do not include bilateral BC and BCs diagnosed when the patient is older than 50 years old in the calculation, and inclusion is restricted to a maximum of three relatives, including the patient. In addition, the tables do not calculate *BRCA1 *and *BRCA2 *probabilities separately.

The descriptive analysis for the two control populations was made using a Gaussian, Poisson, and Gamma linear model for: continuous, count, and percentage variables, respectively. The differences between the group with a pathogenic variant and the group with a neutral variant were obtained using a *t *test, a *z *test, and a *t *test for the Gaussian, Poisson, and Gamma models, respectively. Finally, binary variables were set up as two-by-two tables and the difference between groups was assessed using Fisher's exact test.

A logistic regression was fitted to the pathogenic variations and neutral variants in order to elaborate a predictive model for the pathogenicity of the UVs. The inference criterion used for comparing the models is their ability to predict the observed data; that is, models are compared directly through their minimized minus log-likelihood. When the numbers of parameters in models differed, they were penalized by adding the number of estimated parameters – a form of the Akaike information criterion [23].

Three predictive models (one for variants in both *BRCA1 *and *BRCA2 *and one for each of these separately) were constructed using the best combination of variables that distinguished between deleterious and neutral variants. This was done by first fitting separate univariate models for each clinical feature as well as for BRCAPRO and Myriad scores. To establish which parameters are the most significant ones to predict the pathogenicity of a specific UV, the explanatory variables found to be significant in the univariate analysis are ranked according to their Akaike Information Criterion and are entered accordingly into a new model. This was carried out following a stepwise regression approach.

The receiver operating characteristic (ROC) curves were plotted (data not shown) and the area under the curve (AUC) was calculated for each of the three final models constructed for the control and validation populations.

From a clinical point of view, the most important therapeutic consequences are associated with the assessment of a UV being deleterious. A cutoff point therefore needs to be defined in order to detect the families having a deleterious variant with a high degree of certainty rather than being very sensitive and therefore less specific. The minimum ROC distance ((Sp-1)^2 ^+ (1-Se)^2^) is calculated from the control populations for each final model obtained from the stepwise regression as the cutoff point. Families with a probability value situated above the cutoff point are then predicted to have a deleterious variant with high degree of certainty. This does not, however, necessarily mean that the deleterious variant predicted is the UV identified in that family. Conversely, no prediction can be made as to whether a family has a deleterious or a neutral variant if the value obtained lies under the cutoff point.

When a UV is present in several families, a prediction can be made about whether that UV found is deleterious with more certainty than if it is present in a single family. In order to perform a classification at the UV level, two additional probabilities were computed from the model predictions. The first was the probability that at least one prediction was correct: $1-\prod_{i}\left(1-{\text{P}}_{i}\right)$ where *P*_*i *_is the obtained predicted probability for family *i *of the UV under consideration. The second probability to be computed (which will be referred to as the threshold) is similar but replaces the predicted probabilities by the corresponding cutoff value: 1-(1-CO)^*n *^where CO is the cutoff probability and *n *is the total number of families with the variant under consideration. The variant under consideration is then classified as deleterious if the first probability computed is above the threshold.

In the case of a variant with a single family, the model comes back to comparing the predicted probability with the cutoff point. A conclusion should therefore be made with great care in such cases.

All statistical analyses presented were performed using the freely available program R [24] and the publicly available library 'gnlm' [25].

## Results

### Model building

To build a predictive model, a series of 115 unrelated probands with a pathogenic variant (that is, a mutation) in *BRCA1 *(*n *= 65) or in *BRCA2 *(*n* = 50) are compared with those of a series of 38 unrelated probands with a neutral variant (that is, polymorphism) in *BRCA1 *(*n* = 20) or in *BRCA2 *(*n* = 18). Three models are constructed. A model is first fitted for both *BRCA1 *and *BRCA2 *together, and then for each of these separately (see Table 3).

**Table 3:** Model building steps

	Number of parameters	Akaike information criterion		
		
		Both *BRCA1 *and *BRCA2*	*BRCA1*	*BRCA2*
Intercept	1	86.76	47.38	40.30
Sex	2	*86.60*	48.11	40.35
Proband breast cancer or ovarian cancer	2	87.75	48.37	41.22
Proband breast cancer	2	87.65	48.16	41.30
Proband bilateral breast cancer	2	87.62	48.32	41.06
Proband ovarian cancer	2	*85.49*	*46.84*	40.57
Family affected	2	*86.19*	*45.05*	41.29
Number of first-degree and second-degree relatives^a^	2	87.45	47.93	41.26
Total number of family members affected^a^	2	*85.13*	47.99	*38.40*
Proportion of family members affected^a^	2	86.99	48.30	40.32
Total number of tumors (including bilateral)	2	*83.58*	47.93	*36.24*
Total number of breast tumors	2	87.46	47.82	*38.90*
Number of persons with bilateral cancer	2	87.23	48.17	*38.61*
Total number of ovarian tumors (tnot)	2	*74.76*	*38.08*	*38.04*
Number of persons with ovarian and/or breast tumors (nbot)	2	83.17	46.35	*38.29*
BRCAPRO score	2	**67.36**	**35.90**	**33.74**
Myriad score	2	*69.17*	*36.84*	*34.25*
Age at diagnosis (diag)	2	*82.38*	*42.44*	40.72
BRCAPRO + Myriad	3	66.30	35.55	33.30
BRCAPRO + Myriad + tnot	4	64.58	34.32	-
BRCAPRO + Myriad + tnot + diag	5	***62.46***	29.10	-
BRCAPRO + Myriad + tnot + diag + BRCAPRO:diag	6	-	28.29	-
BRCAPRO + tnot + diag + BRCAPRO:diag	5	-	***27.51***	-
BRCAPRO + Myriad + nbot	4	-	-	***33.16***

Bold numbers represent the univariate and multiple regression models with the lowest Akaike information criterion. Italic numbers represent univariate regressions models with Akaike information criterion lower than that of the corresponding model. ^a^Affected families only.

#### Model for *BRCA1*

The model contains the BRCAPRO1 score (bp1), the total number of ovarian tumors (tnot), the age at diagnosis (diag), and the interaction between BRCAPRO1 and the age at diagnosis:

$$\frac{1}{1\;+\text{ exp}(-(-12.34-2.49\;\times \text{ logit}(\text{bp}1)-3.09\;\times \text{ tnot }+\;0.24\;\times \text{ diag }+\;0.04\;\times \text{ logit}(\text{bp}1)\;\times \text{ diag}))}$$

where $\text{logit}(\text{x})=\text{log}\left(\frac{\text{x}}{1-\text{x}}\right)$.

The highest specificity to predict whether a UV is deleterious that could be obtained with the *BRCA1 *model was 90%, which corresponds to a probability of 0.469 (see Figure 1). The AUC of the ROC curve for the *BRCA1 *model was 0.935, and the lower and upper 95% confidence interval boundaries were respectively 0.91 and 0.96.

#### Model for *BRCA2*

The model contains the BRCAPRO2 score (bp2), the Myriad score (myr), and the number of persons with both ovarian tumors and/or breast tumors (nbot):

$$\frac{1}{1\;+\text{ exp}(-(-3.33-0.57\;\times \text{ logit}(\text{bp2})-0.57\;\times \text{ logit}(\text{myr})\text{ -10}\text{.99 }\times \text{ nbot}))}$$

where $\text{logit}(\text{x})=\text{log}\left(\frac{\text{x}}{1-\text{x}}\right)$.

The highest specificity to predict whether a UV is deleterious that could be obtained with the *BRCA2 *model was 89%, which corresponds to a probability of 0.45 (see Figure 2). The AUC of the ROC curve for the *BRCA2 *model was 0.836, and the lower and upper 95% confidence interval boundaries were respectively 0.784 and 0.887.

### Model validation

**Figure 3:**
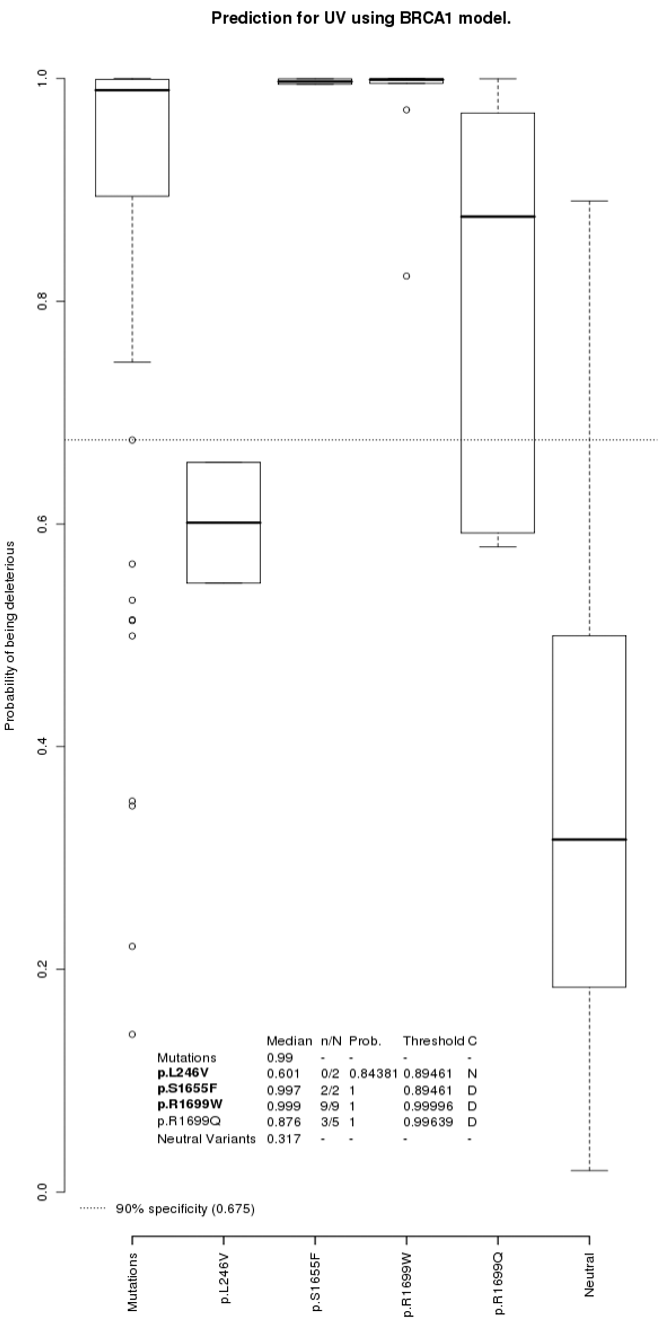
Predicted probabilities and classification of the *BRCA1 *unclassified variants from this study. Box-plots for the *BRCA1 *unclassified variants (UVs) along with the control groups of deleterious and neutral variants. Dotted cutoff lines, probability from the corresponding model that minimizes the receiver operating characteristic distance. The median of each UV and of the control groups (mutations and neutral variants) are presented below. In addition, the number of families above the cutoff point and the total number of families (*n*/*N*) is presented along with the probability of having at least one correct prediction (Prob.) and the probability if all families of the UV under consideration were on the cutoff point (threshold). Finally, the classification (C) as a deleterious variant (D) or not known (N) is also presented. The UVs that have been reported to be either deleterious or neutral in the literature are displayed in bold.

**Figure 4:**
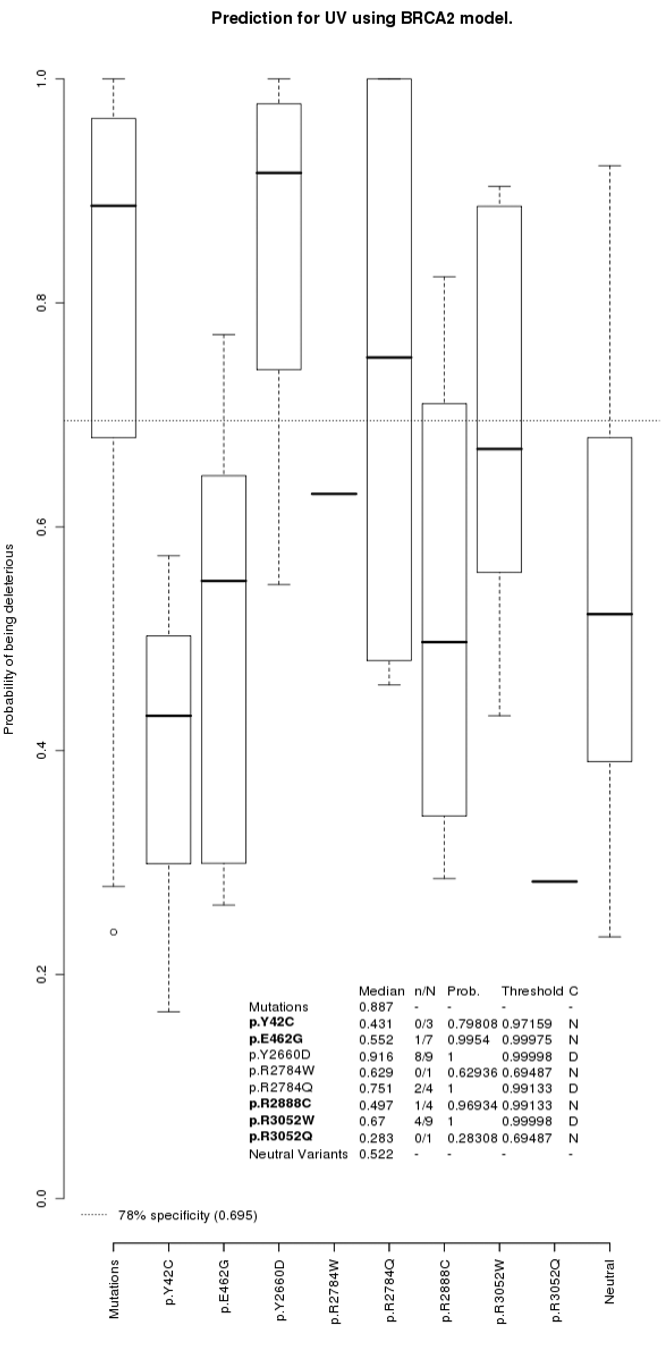
Predicted probabilities and classification of the *BRCA2 *unclassified variants from this study. Box-plots, probability values and classification of the *BRCA2 *unclassified variants (UVs), as explained in Figure 3.

Model validation was performed with the UVs from the present study that had been classified in the literature. From the 12 UVs included in the study, published information about the UV being either deleterious or neutral has become available in the meantime for eight of them: p.L246V, p.S1655F, and p.R1699W in *BRCA1*, and p.Y42C, p.E462G, p.R2888C, p.R3052W and p.R3052Q in *BRCA2 *(see Table 2). We used this information to validate our logistic regression models. The classification as deleterious or not known was therefore computed from the appropriate model for each of these UVs, as shown in Figures 3 and 4.

Amongst the UVs in *BRCA1*, the two families with the p.L246V variant have predicted probabilities below the cutoff point. The families with the p.S1655F and p.R1699W variants are all predicted above the cutoff point (Figure 3). When computing their probabilities (explained above in Materials and methods), the p.S1655F and p.R1699W variants are classified as being deleterious (that is, their probabilities lie above the thresholds) – as opposed to the p.L246V variant, which cannot be classified (that is, probability below the threshold) (see Figure 3). This classification matches previously published results (Table 2). The AUC of the ROC curve for the *BRCA1 *model is 1.000.

Amongst the UVs in *BRCA2*, all families belonging to the p.Y42C and p.R3052Q variants have predicted probabilities below the cutoff point. For both the p.E462G and p.R2888C variants, only one family is predicted above the cutoff point; and for the p.R3052W variant, four out of the nine families are predicted above the cutoff point (Figure 4). When comparing their probabilities, the p.R3052W variant is classified as being deleterious – whereas the p.Y42C, p.E462G, p.R2888C, and p.R3052Q variants cannot be classified. This also matches previously published results (see Table 2). The AUC of the ROC curve for the *BRCA2 *model is 0.789 (95% confidence interval = 0.693 to 0.884).

### Classification of unknown variants from the present study

Three out of the five families with the p.R1699Q variant in *BRCA1 *had predicted probabilities above the cutoff point. This UV was classified as deleterious (Figure 3).

The families with the p.Y2660D in *BRCA2 *had the highest median of all the *BRCA2 *UVs from this study (median = 0.916), with eight of the nine families predicted above the cutoff point. The computed probabilities classified this UV as being deleterious (Figure 4).

The p.R2784W variant in *BRCA2 *could not be classified because the only family with this variant was predicted below the cutoff point (Figure 4).

Finally, two out of the four families with the p.R2784Q variant in *BRCA2 *had predicted probabilities above the cutoff point. This UV was classified as deleterious (Figure 4).

## Discussion

### About the models

Registration of BC and/or OC events in a family is easy to perform and is the most direct tool to assess the clinical significance of a genetic variation. In the present study we developed logistic regression models with the best combination of clinical features that distinguish families with deleterious variants from those with neutral variants, and applied them to assess the pathogenicity of 12 UVs found in 59 Dutch families.

To estimate which families with a UV have features similar to those with a proven deleterious mutation, we chose probands with neutral variants as negative controls. In the study of Easton and colleagues, the negative controls were probands with a wild-type genotype [14]. Although the size of the negative control population would have been larger with the latter population, we consider a population with rare neutral variants to be a better negative control to classify UVs, which are also rare variants.

The BRCAPRO and Myriad II scores are useful tools for calculating the probability of finding a pathogenic variant [26-31], as well as for distinguishing deleterious variants from UVs as a group [15]. In the present study we show that these scores are also useful for the classification of individual UVs. Our model for *BRCA1 *performs better than the one for *BRCA2 *to predict the deleterious effect of UVs, which is in line with the reduced penetrance of the *BRCA2 *pathogenic variants. Accordingly, Kang and colleagues [30] and James and colleagues [31] have also reported that the BRCAPRO and Myriad models perform better for predicting *BRCA1 *than *BRCA2 *pathogenic variants.

By testing UVs that are present in multiple families, as is the case for most of the UVs in the present study, the effect of possible confounders linked to a particular family can be overcome. Confounders can result in either high or low false probabilities. A false low probability can occur when the BRCAPRO and Myriad II models are not able to incorporate important information about the cancer history in a particular family (for instance, if there is no information about relatives or if the affected relatives are only third-degree relatives). Conversely, when the BRCAPRO and Myriad models adequately reflect the cancer history of the families with low scores, one has to think of a possible confounder whenever the high score in one of the families is discordant with the rest for a particular UV. In those cases, information about co-segregation with the disease can give the answer as to whether the UV found is the actual cause of the disease in that/those particular family(ies) or the high scores are the result of another, as yet unidentified, deleterious mutation. Indeed, the sensitivity of genetic testing has been estimated to be at least 85%, with false negatives including mutations of as yet unidentified cancer genes [26]. To account for the possibility that a mutation has escaped detection, therefore, we recommend that more than a single family with the same variant has to be available in order to be able to classify the variant under consideration.

### About the unclassified variants

#### Unclassified variants from the validation set

From the UVs included in the validation set, the p.S1655F and p.R1699W variants in *BRCA1 *and the p.R3052W variant in *BRCA2 *were classified as deleterious with our model. Abkevich and colleagues [5] and Glover [32] have also reported on the p.S1655F variant and considered it deleterious. The p.R1699W *BRCA1 *variant has already been classified as deleterious in the Breast Cancer Information Core [1]. The p.R3052W variant of *BRCA2 *has also been recently classified as deleterious based on a functional assay that measures the DNA-repair function by homologous recombination [33].

Conversely, the remaining five UVs – p.L246V in *BRCA1*, and the p.Y42C p.E462G, p.R3052Q, and p.R2888C variants in *BRCA2 *– could not be classified. The fact that all five variants have been reported to be neutral variants in the literature [5-7,9,11-14] validates the sensitivity and specificity of our models.

#### Classification of the unknown variants

The p.R1699Q *BRCA1 *variant was classified as deleterious according to our model. Earlier attempts to classify this particular UV have not lead to a uniform conclusion [5,6,9,12,32,34,35]. Abkevich and colleagues [5] consider it to be deleterious, whereas for Goldgar and colleagues [6], Chenevix-Trench and colleagues [9], Glover [32], and Clapperton and colleagues [34] the R1699Q genetic variation remains of uncertain significance. Vallon-Christersson and colleagues [35] find a discrepancy in the transactivation activity depending on the type of cells transfected with this UV: yeast cells (neutral) or mammalian cells (deleterious). For Lovelock and colleagues, this variant has low to moderate risk of being pathogenic based on functional analysis; that is, p.R1699Q appeared defective in nuclear foci formation using trypsin sensitivity analysis as a result of BRCA C-terminal region destabilization [12]. By referring to it as low to moderate risk, the authors imply that genetic variations can have different degrees of pathogenicity (that is, penetrance) [12]. We agree with this hypothesis – that certain missense genetic variations may have a milder effect than stop-codon variations, and therefore show intermediate features. This hypothesis may explain the discordant conclusions among the different studies about this UV (and possibly others as well) of being either deleterious or neutral. In the case of this particular UV, a factor of uncertainty is also the lack of co-segregation in one family.

The p.Y2660D variant in *BRCA2 *is considered deleterious according to our model. This UV has not been studied before. In addition, this UV showed full co-segregation in the five families studied and it affects a highly conserved amino acid, which also corroborate that this UV is deleterious.

The p.R2784Q variant in *BRCA2 *is also considered deleterious according to our model. This UV has not been classified before. From a biochemical point of view, arguments in favor of causality are that the arginine at that position is highly conserved and that the amino acid substitution causes a polarity change.

Neither the predicted probabilities nor the limited number of families allowed definitive conclusions to be made about the p.R2784W variant. Functional studies performed for this variant were also inconclusive [33].

## Conclusions

We have identified a combination of variables from the cancer history of the probands and their families that significantly distinguish families with proven deleterious variants from those with neutral variants, and we have used them to develop logistic regression models to classify individual UVs in the *BRCA *genes. We used these models to classify a selected group of 12 UVs, the majority present in multiple families in the Netherlands. Using those models the p.S1655F and p.R1699W variants in *BRCA1 *were classified as deleterious, which corroborates previous literature reports. According to our model, the p.R1699Q variant is also classified as deleterious – but previous reports about this UV have been contradictory. The p.Y2660D and p.R2784Q variants in *BRCA2*, which have not been reported before, were also classified as deleterious. From the six UVs that could not be classified, five (the p.L246V variant in *BRCA1*, and the p.Y42C, p.E462G, p.R2888C, and p.R3052Q variants in *BRCA2*) have been reported in the literature as being neutral variants. The p.R2784W variant in *BRCA2 *remains uncertain.

Since the parameters evaluated are readily available, we consider those developed models a useful tool to evaluate missense variants in the clinical genetic practice. Moreover, because those parameters can be evaluated in families with all types of UVs, those models are potentially suitable for the classification of all types of UVs.

## Abbreviations

AUC: area under the curve; BC: breast cancer; bp1: BRCAPRO1 score; bp2: BRCAPRO2 score; *BRCA*: breast cancer gene; diag: age at diagnosis; myr: Myriad score; nbot: number of persons with ovarian and/or breast tumors; OC: ovarian cancer; ROC: receiver operating characteristic; tnot: total number of ovarian tumors; UV: unclassified variant.

## Competing interests

The authors declare that they have no competing interests.

## Authors' contributions

EBGG designed the study, participated in the inclusion of patients and controls, and drafted the manuscript. JCO contributed substantially to the drafting of the manuscript, and together with CJvA, NH, RO, SV, CJD, MGEMA, and TAMvO provided the patients for the study population. MT retrieved the information from the patients' records included in the control populations and created the database for the study. MJB provided the information about the variants used in the control study, and together with AHvdH, ML, AvdO, RBvdL, JTW, and JJPG provided information about the UVs analyzed in their laboratory and provided co-segregation data. PJL carried out all of the statistical analyses performed in this study. MPGV secured funding for the project and elaborated the list of UVs to be analyzed for this study, and together with FBLH and PD participated in project conception and critical review of the manuscript.
